# Reptile-associated *Borrelia* species in the goanna tick (*Bothriocroton undatum*) from Sydney, Australia

**DOI:** 10.1186/s13071-017-2579-5

**Published:** 2017-12-20

**Authors:** Jessica L. Panetta, Radek Šíma, Nichola E. D. Calvani, Ondřej Hajdušek, Shona Chandra, Jessica Panuccio, Jan Šlapeta

**Affiliations:** 1 Sydney School of Veterinary Science, Faculty of Science, University of Sydney, Sydney, NSW 2006 Australia; 2 Institute of Parasitology, Biology Centre of the Czech Academy of Sciences, Branišovská 31, 37005 České Budějovice, Czech Republic

**Keywords:** *Bothriocroton undatum*, *Borrelia*, Goanna tick, DNA extraction, MiSeq, Illumina, NGS, Ixodidae, *Coxiella burnetii*

## Abstract

**Background:**

Knowledge on the capacity of Australian ticks to carry *Borrelia* species is currently limited or missing. To evaluate the potential of ticks to carry bacterial pathogens and their DNA, it is imperative to have a robust workflow that maximises recovery of bacterial DNA within ticks in order to enable accurate identification. By exploiting the bilateral anatomical symmetry of ticks, we were able to directly compare two DNA extraction methods for *16S* rRNA gene diversity profiling and pathogen detection. We aimed to assess which combination of DNA extraction and *16S* rRNA hypervariable region enables identification of the greatest bacterial diversity, whilst minimising bias, and providing the greatest capacity for the identification of *Borrelia* spp.

**Results:**

We collected Australian endemic ticks (*Bothriocroton undatum*), isolated DNA from equal tick halves using two commercial DNA extraction methods and sequenced samples using V1-V3 and V3-V4 *16S* rRNA gene diversity profiling assays. Two distinct *Borrelia* spp. operational taxonomic units (OTUs) were detected using the V1-V3 *16S* rRNA hypervariable region and matching *Borrelia* spp. sequences were obtained using a conventional nested-PCR. The tick *16S* rRNA gene diversity profile was dominated by *Rickettsia* spp. (98–99%), while the remaining OTUs belonged to Proteobacteria (51–81%), Actinobacteria (6–30%) and Firmicutes (2–7%). Multiple comparisons tests demonstrated biases in each of the DNA extraction kits towards different bacterial taxa.

**Conclusions:**

Two distinct *Borrelia* species belonging to the reptile-associated *Borrelia* group were identified. Our results show that the method of DNA extraction can promote bias in the final microbiota identified. We determined an optimal DNA extraction method and *16S* rRNA gene diversity profile assay that maximises detection of *Borrelia* species.

**Electronic supplementary material:**

The online version of this article (doi: 10.1186/s13071-017-2579-5) contains supplementary material, which is available to authorized users.

## Background

Ticks are known to have the ability to transmit bacterial, viral and protozoal agents during blood feeding episodes, making them one of the most important arthropod vectors parasitising reptilian, avian and mammalian species, including humans [1–3]. In Australia, *Ixodes holocyclus* and *Bothriocroton hydrosauri* can carry and transmit *Rickettsia australis* and *Rickettsia honei*, respectively, causing rickettsiosis in humans [4–7]. More recently, a novel species of *Candidatus* Borrelia tachyglossi was identified in *Bothriocroton concolor* and *I. holocyclus* ticks collected from the short-beaked echidna (*Tachyglossi aculeatus*) [8, 9]. *Borrelia* species and their associated human illnesses such as Lyme disease, have been a highly debated and controversial topic for over 25 years in Australia [10–12]. To bring a new line of evidence to the debate on Lyme disease and other human tick-borne diseases, a complete map of bacteria in Australian ticks is needed [9, 13, 14]. Lyme disease agents [spirochetes *Borrelia burgdorferi* (*sensu lato*)] are not known to be transmitted locally in Australia, but Lyme-like disease caused by unknown pathogens are considered to exist [10–12, 15, 16]. Baseline data and a consensus of potentially pathogenic bacteria in Australian ticks are imperative to initiate inquiry into the existence of the bacterial causality of human disease. To evaluate the potential of ticks to carry bacterial pathogens, specifically bacterial DNA, it is imperative to obtain a high quality and quantity of bacterial DNA in order to enable unbiased tick surveys.

Next-generation sequencing (NGS) technologies have seen a shift in tick microbial studies from targeting individual bacterial species, to sequencing whole tick assemblages [17, 18]. However, a universal consensus on the optimal DNA extraction protocol for hard tick species is still lacking: a significant issue when the hard chitinous exoskeleton and multiple life stages of ixodid ticks are considered [4, 6, 7, 9, 19, 20]. DNA extraction is a critical component of microbiota studies, due to its influence on the abundance and diversity of bacterial species identified [21–23]. Determining the bias of DNA extraction methods towards or away from certain bacterial groups is confounded by variability introduced before the DNA is even extracted, due to the heterogeneity of the individual tick, and their small size [18, 24, 25]. Thus, there is a need to determine the bias of DNA extraction methods on a homogenous tick population.

The aim of this study was to assess which combination of DNA extraction method with *16S* ribosomal (rRNA) hypervariable region through NGS sequencing gave the greatest bacterial diversity and enabled the identification of *Borrelia* spp. and *Rickettsia* spp. To directly compare pre-sequencing methodologies, we exploited the symmetrical body plan of ticks [26]. Our approach involved longitudinally bisecting tick samples (*Bothriocroton undatum*) and processing each assumed identical half with a different DNA extraction method. Our workflow allowed us to determine and associate microbiome variability with the method and assay applied. We were then able to confirm our workflow using conventional PCR techniques targeting *Borrelia* spp. and *Rickettsia* spp.

## Methods

### Collection and identification of ticks

A total of 25 ticks (Tick1–25) were collected from a wild lace monitor (*Varanus varius*) in Terrey Hills, New South Wales, Australia in December 2016 (Additional file 1: Table S1). The lace monitor was brought to the attention of the veterinary surgeon by a member of the public, and was euthanized by the registered veterinarian due to suspected rat poisoning and emaciation. Ticks were collected opportunistically post-mortem, immediately submerged in 70% (*w*/*v*) ethanol and submitted to the Sydney School of Veterinary Sciences (SSVS), The University of Sydney. Ticks were stored at -20 °C until morphological identification and DNA isolation. Ticks were morphologically identified with the aid of a stereomicroscope (Olympus, Macquarie Park, Australia), using dichotomous keys and character matrices [26, 27].

### Bisecting ticks for comparison of DNA extraction methods

Prior to DNA isolation, individual ticks were surface sterilised [28]; 1 min submerged in 3% hydrogen peroxide (H_2_O_2_), 30 s submerged in 70% (*w*/*v*) ethanol and 2 min submerged in phosphate buffered saline (PBS, pH = 7.4).

**Fig. 1:**
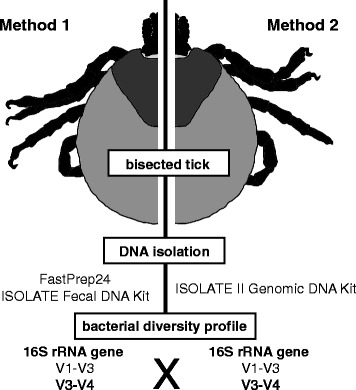
Summary of a workflow taking advantage of the symmetrical body plan of ticks. All tick samples were longitudinally bisected, each half being subjected to DNA extraction by either the ISOLATE Fecal DNA kit (Method 1) or the ISOLATE II Genomic DNA Kit (Method 2). All samples were sequenced using gene diversity profiling assays, targeting both the V1-V3 and V3-V4 *16S* rRNA hypervariable regions

Tick1 to Tick6 (*n* = 6) were chosen based on level of engorgement and size (fully engorged, uniformly ~1 cm in width). Each tick was longitudinally bisected using a sterile disposable scalpel blade (Fig. 1) and each half was subjected to a different DNA extraction method. Method 1 exploited a mechanical maceration protocol where the first half of each tick was placed in a 1.5 ml bead-beater with ceramic beads and homogenised using a high-speed benchtop homogeniser FastPrep-24 (MP Biomedicals, Seven Hills, Australia) for 40 s at 6.0 m/s, followed by extraction using the ISOLATE Fecal DNA Kit (Bioline, Eveleigh, Australia) as per the manufacturer’s instructions. Method 2 consisted of enzymatic cell lysis followed by the ISOLATE II Genomic DNA Kit (Bioline) as per the manufacturer’s instructions, with overnight (16 h) digestion with Proteinase K. To monitor DNA isolation efficiency, 5 μl of DNA Extraction Control 670 (Bioline) was included in each sample, regardless of the DNA extraction method applied. DNA quality and quantity was further confirmed using a Qubit dsDNA HS assay on the Qubit® 2.0 Fluorometer (Thermo Fisher Scientific, North Ryde, Australia). DNA was stored at -20 °C until PCR amplification. DNA Extraction Control 670 (Bioline) served to validate the success of the extraction step and was amplified as per the manufacturer’s protocol and instructions.

Tick7 to Tick25 (*n* = 19) were surface sterilised, longitudinally bisected and both halves were together subjected to Method 1 for DNA extraction and processed as described above.

### Amplification of the tick’s cytochrome *c* oxidase subunit 1

A 658 nucleotide (nt) fragment of the cytochrome *c* oxidase subunit 1 (*cox*1) gene was amplified using PCR with forward primer S0725 (F1) (5′-TAC TCT ACT AAT CAT AAA GAC ATT GG**-**3′) and reverse primer S0726 (R1) (5′-CCT CCT CCT GAA GGG TCA AAA AAT GA-3′) (primers as published in O. Kushimo thesis ‘The tick genus *Amblyomma* in Africa: Phylogeny and multilocus DNA barcoding’ from Georgia Southern University, 2013; primers cited as M. Montagna, unpublished data). MyTaq™ Red Mix (Bioline) was used for 30 μl reactions with 2 μl of DNA template in a T100 Thermal Cycler (BioRad, Gladesville, Australia) as previously described [29]. PCR products were bi-directionally sequenced by Macrogen Ltd. (Seoul, South Korea). Sequences were assembled and aligned using CLC Main Workbench 6.9.1 (CLC bio, Denmark). The evolutionary history of tick *cox*1 gene sequences was inferred using the Minimum Evolution (ME) method, with model selection based on maximum likelihood in MEGA7 (v.7.0.14) [30]. Bootstrap support was calculated as a percentage of replicate trees in which the associated taxa clustered together as implemented in MEGA7.

### Next-generation sequencing of bacterial *16S* rRNA gene amplicons

Tick DNA samples (Tick1–6) were subjected to *16S* rRNA gene diversity profiling assays at the Australian Genome Research Facility (Brisbane, Australia). Sequencing of the V1-V3 and V3-V4 *16S* rRNA hypervariable regions was performed on the Illumina Miseq (300-nt pair-end) using the following assay; 16S (V1-V3): 27F (5′-AGA GTT TGA TCM TGG CTC AG-3′) with 519R (5′-GWA TTA CCG CGG CKG CTG-3′) and 16S (V3-V4) 341F (5′-CCT AYG GGR BGC ASC AG-3′) with 806R (5′-GGA CTA CNN GGG TAT CTA AT-3′). Paired-end reads were assembled using PEAR (version 0.9.5). Primers were identified and trimmed. Trimmed sequences were processed using Quantitative Insights into Microbial Ecology (QIIME 1.8/1.9.1), USEARCH (v.8.0.1623) and UPARSE software. Sequences were quality filtered and full length duplicate sequences were removed and sorted by abundance using USEARCH. Singletons or unique reads in the data set were discarded. Sequences were clustered followed by chimera filtering using the “rdp gold” database as a reference. To obtain the number of reads in each OTU, reads were mapped back to OTUs with a minimum identity of 97%. Using QIIME, taxonomy was assigned using the Greengenes database 5 (version 13 8, Aug 2013). Unassigned OTUs were excluded as well as any belonging to mitochondria and chloroplasts.

### Multivariate statistical analysis of tick microbiome

Multivariate analytical procedures were used to investigate patterns of variation in the composition of bacterial assemblages among tick samples. The microbiome abundance matrix with taxonomy of each OTU and sample associated factors were imported for multivariate statistical analysis in PRIMER v.7 [31]. Data from V1-V3 and V3-V4 *16S* rRNA hypervariable regions were analysed separately. Each sample was associated with the following factors: method and tick number. The data matrix of OTU abundances (or chosen taxonomical level abundance) was fourth-root-transformed. A reasonably severe transformation was appropriate in order to reduce the contribution of the most abundant taxonomical categories (OTU, species, genera, etc.). Variation in the structure of bacteria within the ticks was examined using Bray-Curtis resemblance measures. An analysis of similarities (ANOSIM [32]) was used to test the null hypothesis of no differences among the communities of defined groupings (significance level, *P* = 0.05). Non-metric multidimensional scaling (nMDS) ordination [33] was undertaken to visualize and explore the patterns of community similarities amongst all samples. The goodness-of-fit of the resulting two dimensional nMDS plot was measured using Kruskal’s stress formula I [33]. Visualisation was enhanced by marking different factors (e.g. age and parasite status) superimposed on the ordination plots as symbols and numbers to help evaluate their potential effects on bacterial assemblage structure.

Diversity indices including *S*, total number of species (OTUs); *N*, species richness (Margalef); *H′*, Shannon diversity index with logs to the base e; and *1-λ’*, Simpson’s diversity index, were calculated for the OTU tables in PRIMER v.7 [31]. Shannon diversity indices were evaluated using t-tests in Microsoft Excel (2016) to compare diversity estimates between V1-V3 and V3-V4 *16S* rRNA hypervariable regions, and Method 1 and Method 2. Richness was obtained by calculating the number of different taxonomic families and genera found within V1-V3 and V3-V4 *16S* rRNA hypervariable regions, where a threshold of 30 reads was applied. Venn diagrams were constructed in RStudio® (version 1.0.143 RStudio, Inc.) using the ‘VennDiagram’ package [34].

Abundance at different taxonomic levels and bar reads representing uncultured or unknown bacteria were calculated using Microsoft Excel (2016). Abundance measures for each phylum, class, order, family and genus between each assay (Method 1 + V1-V3, Method 1 + V3-V4, Method 2 + V1-V3, Method 2 + V3-V4) were used to further determine the presence of bias. A Tukey’s multiple comparisons test was conducted in GraphPad Prism 7.03 (GraphPad Software Inc. 2017), comparing each measure of bacterial abundance between each of the four combinations.

### Detection of *Borrelia* spp. and *Rickettsia* spp. using conventional nested-PCR, and *Coxiella burnetii* using conventional PCR

Detection of *Borrelia* spp. spirochetes in ticks was performed by two independent conventional nested-PCR assays (*16S* rRNA gene, *23S* rRNA gene). A *16S* rRNA gene nested-PCR amplifying a ~1250-nt fragment was performed on all tick samples (Tick1–25) at SSVS using previously published primers [9]. The *16S* rRNA gene of *Borrelia* spirochetes was amplified in a reaction volume of 30 μl, containing 15 μl of MyTaq Red™ Mix (Bioline), 10 pmol of each primer (Bor-16F (S0778), 5′-TGC GTC TTA AGC ATG CAA GT-3′ / Bor-1360R (S0779), 5′-GTA CAA GGC CCG AGA ACG TA-3′ for the first round; Bor-27F (S0780), 5′-CAT GCA AGT CAA ACG GAA TG-3′ / Bor-1232R (S0781), 5′-ACT GTT TCG CTT CGC TTT GT-3′ for the second round [9]), template (2 μl of purified DNA for the first round and 1 μl aliquot of the first PCR product in the second round), and PCR-grade water. The PCR was run on a T100 Thermal Cycler (Bio-Rad), including initial denaturation at 95 °C for 3 min, followed by 35 cycles of 95 °C for 15 s, 55 °C for 15 s, 72 °C for 30 s, and a final elongation for 5 min at 72 °C for both primary and secondary reactions. Each run included a blank negative control with sterile PCR-grade water. A positive control (*16S* rRNA gene amplification) was excluded to minimise contamination. A *23S* rRNA gene nested-PCR amplifying a 222-nt fragment was performed on Tick1–6 at the Biology Centre of the Academy of Science of the Czech Republic [35]. The *23S* rRNA gene of *Borrelia* spirochetes was amplified in a reaction volume of 25 μl, containing 12.5 μl of FastStart PCR MasterMix (Roche, Praha, Czech Republic), 10 pmol of each primer (Bor-1, 5′-AGA AGT GCT GGA GTC GA-3′ / Bor-2, 5′-TAG TGC TCT ACC TCT ATT AA-3′ for the first round; Bor-3, 5′-GCG AAA GCG AGT CTT AAA AGG-3′ / Bor-4, 5′-ACT AAA ATA AGG CTG AAC TTA AAT-3′ for the second round [35]), template (4 μl of purified DNA for the first round, 1 μl of aliquot for the first PCR product in the second round), and PCR-grade water. The PCR was run on an Applied Biosystems 2720 Thermal Cycler (ThermoFisher Scientific), including initial denaturation at 95 °C for 10 min followed by 40 cycles of 95 °C for 30 s, 53 °C for 30 s, 72 °C for 30 s, and a final elongation for 7 min at 72 °C. The amplification program for the second round was the same, except for the annealing temperature which was 58 °C.

A diagnostic conventional nested-PCR assay targeting the *gltA* gene of *Rickettsia* spp. was applied to Tick1–6 as previously described and adopted [29, 36]. A ~650-nt fragment of *gltA* was amplified in a final reaction volume of 30 μl, containing 15 μl MyTaq Red™ Mix (Bioline), 10 pmol of each primer (gltA-F1 (S0659), 5′-GCA AGT ATT GGT GAG GAT GTA ATC-3′ / gltA-R1 (S0660), 5′-CTG CGG CAC GTG GGT CAT AG-3′ for the first round; gltA-F2 (S0661), 5′-GCG ACA TCG AGG ATA TGA CAT-3′ / gltA-R2 (S0662) 5′-GGA ATA TTC TCA GAA CTA CCG-3′, for the second round), template (2 μl of purified DNA for the first round and 1 μl aliquot of the first PCR product in the second round), and PCR-grade water. The PCR was run on a T100 Thermal Cycler (Bio-Rad), including initial denaturation at 95 °C for 3 min, followed by 35 cycles of 95 °C for 15 s, 55 °C for 15 s, and a final elongation for 5 min at 72 °C for both primary and secondary reactions. Each run included a blank negative control with sterile PCR-grade water.

*Coxiella burnetii* was detected using a conventional PCR targeting IS1111-repetitive elements with IS1111aF (S0797) (5′-GTC TTA AGG TGG GCT GCG TG-3′) and IS1111aR (S0798): (5′-CCC CGA ATC TCA TTG ATC AGC-3′) [37]. MyTaq™ Red Mix (Bioline) was used in 30 μl reactions with 2 μl of DNA template in a T100 Thermal Cycler (BioRad). The cycling conditions were as follows: initial denaturation at 95 °C for 3 min, followed by 34 cycles of 95 °C for 15 s, 55 °C for 15 s, 72 °C for 15 s, and a final elongation for 5 min at 72 °C. The positive control for *C. burnetii* was DNA extracted from the Q Fever vaccine (Q-VAX®) (kindly provided by Jacqui Norris). Sterile PCR-grade water was used as a blank negative control.

PCR products were visualized on 2% (*w*/*v*) agarose gel with GelRed™ (Biotium, Inc., Fremont, CA, USA). Products from the *16S* rRNA gene assay (*Borrelia*) and *gltA* gene assay (*Rickettsia*) were directly and bi-directionally sequenced using amplification primers at Macrogen Ltd. (Seoul, South Korea). Sequences were assembled and chromatographs visually inspected and compared to related sequences using CLC Main Workbench 6.9.1 (CLC bio, Aarhus, Denmark). The evolutionary history of *Borrelia* spp. *16S* rRNA gene sequences was inferred using the Minimum Evolution (ME) method, with model selection based on maximum likelihood in MEGA7 (v.7.0.14) [30]. Bootstrap support was calculated as a percentage of replicate trees in which the associated taxa clustered together as implemented in MEGA7.

### Quantification of bacteria using real-time PCR

Bacterial load was determined using a universal bacterial real-time PCR assay targeting the *16S* rRNA gene [38]. The 10 times dilution of *Escherichia coli* strain ATCC 11775 (kindly gifted by Denise Wigney) suspended cells was used to construct a standard curve. A set of generic bacterial primers (S0775) (5′-TCC TAC GGG AGG CAG CAG T-3′) / (S0776) (5′-GGA CTA CCA GGG TAT CTA ATC CTG TT-3′) and probe (S0777) (5′-CGT ATT ACC GCG GCT GCT GGC AC-3′) labelled with FAM-BHQ1 was used to quantify the amount of bacterial DNA present within each tick sample from Method 1 and Method 2. SensiFAST™ Probe No-ROX Kit (Bioline) was used according to the manufacturer’s instructions. The PCR included 2 μl of DNA template and was run on the CFX96 Touch Real-Time PCR (Bio-Rad), and copy number analysed using the corresponding CFX Manager 3.1 (Bio-Rad) software. Real-time PCR cycling conditions included an initial denaturation step at 95 °C for 3 min, followed by 35 cycles of 95 °C for 10 s and 60 °C for 30 s. An extraction control real-time PCR was performed for each sample to monitor DNA isolation efficiency and PCR inhibitors as described previously. A t-test using Microsoft Excel (2016) was used to calculate significant differences between the average numbers of bacterial cells found using each extraction kit.

## Results

### The goanna tick (*Bothriocroton undatum*) from the lace monitor (*Varanus varius*)

**Fig. 2:**
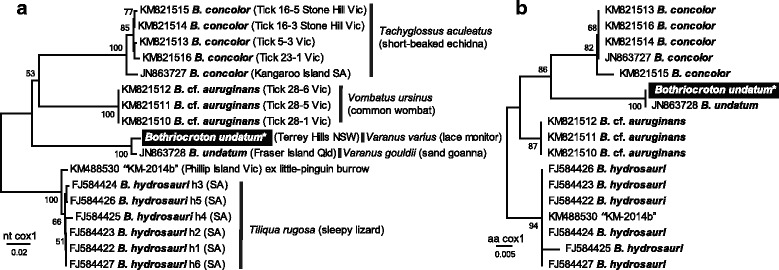
Phylogenetic position of the Goanna tick (*Bothriocroton undatum*). Ticks were collected from a wild lace monitor (*Varanus varius*) from Sydney, Australia. **a** The evolutionary history based on *cox*1 nucleotide (nt) sequences was inferred using the Minimum Evolution (ME) method with Kimura-2 parameter model and percent bootstrap support percentages (200 replicates) shown next to the branches was calculated in MEGA7. There were a total of 521 positions in the final dataset. **b** The evolutionary history based on *cox*1 amino acid (aa) sequences was inferred using the ME method, with bootstrap support percentages test (200 replicates) shown next to the branches. The evolutionary distances were computed using the Poisson correction method and are in the units of the number of aa substitutions per site. There were a total of 173 positions in the final dataset. Accession number with tick species name, tick identifier and locality in Australia is followed by host name. *All *B. undatum* ticks (*n* = 6, Tick1 to Tick6) had identical *cox*1 sequences

All ticks (*n* = 25) collected from the lace monitor (*Varanus varius*) were morphologically identified as females from the species *B. undatum*. Amplification and DNA sequences of *cox*1 mtDNA from the DNA of six tick specimens demonstrated close identities with the *cox*1 sequences of *B. undatum* (98.9%, 650/658, JN863728). All eight nucleotide substitutions were synonymous, revealing 100% (219/219) identity with the *cox*1 amino acid sequence of *B. undatum* (JN863728). Multiple sequence alignment with related *Bothriocroton* spp. sequences at either nucleotide or amino acid sequence confirmed conspecificity with *B. undatum* (Fig. 2).

The real-time PCR assay estimated the average number of bacterial cells for Method 1 and Method 2 to be 48,139 and 148,660 per entire tick, respectively (Tick 1–6). The results of the t-test demonstrated a significant increase (paired t-test, *t*_(5)_ = 8.936, two-tailed *P* = 0.0003) in the number of bacterial cells detected in DNA extracted using Method 2 compared to Method 1. DNA extraction control was amplified for examined ticks, confirming successful and uninhibited DNA isolation. The Qubit dsDNA HS assay found the average quantity of DNA for Method 1 and Method 2 to be 21 ng/μl and 51 ng/μl, respectively (Additional file 1: Table S2).

### The greater tick microbiome diversity using a combination of *16S* rRNA assays

**Fig. 3:**
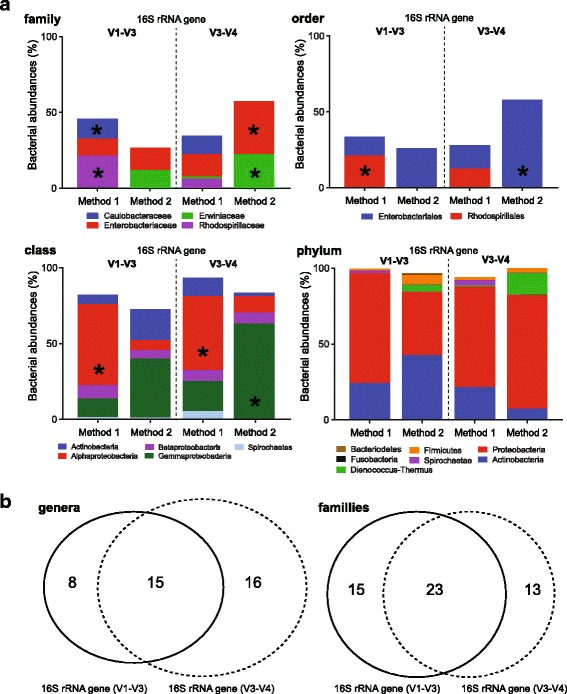
Bacterial diversity profiles of the Goanna tick (*Bothriocroton undatum*). Bacterial abundance and bias at family, order, class and phylum levels were retrieved from a combination of different workflows that included V1-V3 or V3-V4 *16S* rRNA gene diversity profiles assays, and either Method 1 or Method 2 for the Goanna tick (*Bothriocroton undatum*). **a** Graphs with bacterial families and orders show only those with significant bias between the workflows. The graph depicting bacterial classes demonstrates the five most dominant bacterial classes found in each of the workflows, including spirochetes (*Borrelia* spp.). The graph of bacterial phyla shows no bias found between any of the workflows. Bias between bacterial abundances at different taxonomical levels was evaluated using Tukey’s multiple comparisons test, (*P* < 0.05) and is indicated with ‘*’. **b** Venn diagrams representing the number of taxonomic families and genera identified within each *16S* rRNA hypervariable region as a measure of richness. A threshold of 30 reads and 97% identification was applied

**Fig. 4:**
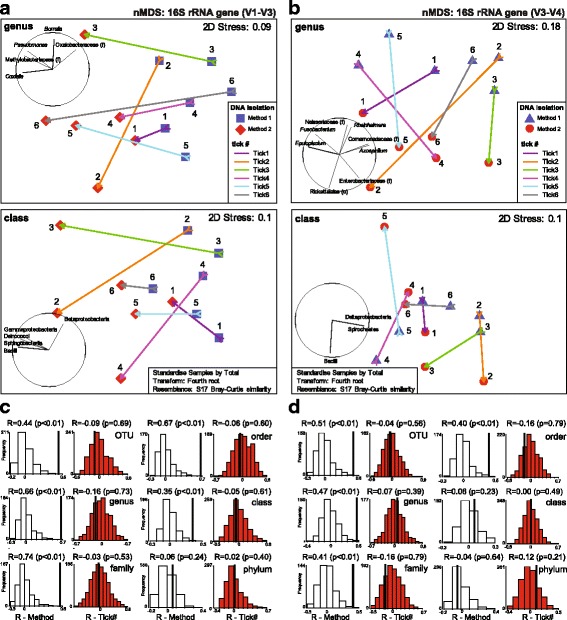
Multivariate analysis of the bacterial profiles of the Goanna tick (*Bothriocroton undatum*). Non-metric MDS ordination (nMDS) plots for bacterial abundance and analysis of similarity (ANOSIM). The ordinations are from abundances at the genus or class level of (**a**) 124 OTUs (V1-V3 *16S* rRNA gene diversity profiling assay) and (**b**) 126 OTUs (V3-V4 *16S* rRNA gene diversity profiling assay), with resulting stress values of (**a**) 0.09 (genus), 0.10 (class) and (**b**) 0.18 (genus), 0.10 (class). Superimposed are vector plots for taxa (correlation > 0.8) displaying observed responses to the gradient [direction reflects the Pearson correlation of transformed abundances, length represents the multiple correlation coefficient from the linear regression on the ordination points (circle is a correlation = 1)]. Individual halved ticks are joined by solid lines to identify the community differences using nMDS. Histograms of permutated distributions of the test statistics R (up to 999 permutations, ANOSIM; null hypothesis - is no significant difference among communities, *P* < 0.05) with observed R (bold) at different taxonomic level evaluated for DNA isolation method (transparent) and tick (red) (**c**, **d**)

Ticks (*n* = 6, Tick1–6) were longitudinally bisected. DNA from each half was isolated with either Method 1 or Method 2, and a microbiome profile was generated using V1-V3 (*n* = 12) and V3-V4 (*n* = 12) *16S* rRNA gene diversity profiling assays (Figs. 1 and 3, Additional file 1: Table S3). The V1-V3 *16S* rRNA gene diversity profiling assays yielded 1,112,938 raw reads that were quality filtered into 834,157 (min. 19,420; max. 114,048; *n* = 12) high quality reads (excluding singletons) and clustered into 126 bacterial OTUs. The V3-V4 *16S* rRNA gene diversity profiling assays yielded 1,895,826 raw reads that were quality filtered into 1,602,905 (min. 40,128; max. 225,303; *n* = 12) high quality reads (excluding singletons) and clustered into 124 bacterial OTUs. Both V1-V3 and V3-V4 *16S* rRNA gene diversity profiles using either method (Fig. 1) were dominated by OTUs belonging to the genus *Rickettsia* (98–99%). Excluding the dominating OTU (OTU_1; *Rickettsia*), reads belonging to the phyla Proteobacteria (51–81%), Actinobacteria (6–30%) and Firmicutes (2–7%) dominated both hypervariable regions (Fig. 3a). *16S* rRNA gene diversity profiles from both Method 1 and Method 2 were dominated by Alphaproteobacteria (49–53%) and Gammaproteobacteria (39–63%) respectively (Fig. 3a). OTUs assigned to *Borrelia* spp. accounted for ~7% of bacterial reads between the two hypervariable regions, and *Coxiella* spp. accounted for 1.2% of bacterial reads. Shannon diversity indices (*H′*) demonstrated no significant difference in the level of bacterial diversity between V1-V3 and V3-V4 *16S* rRNA gene diversity profiling assays (paired t-test, *t*_(5)_ = 1.4561, *P* > 0.05; paired t-test *t*_(5)_ = 0.6848, *P* > 0.05), or between Method 1 and Method 2 (paired t-test, *t*_(5)_ = 0.1419, *P* > 0.05; paired t-test *t*_(5)_ = 1.3524, *P* > 0.05) (Table 1). A large proportion of bacterial genera and classes were unique to either the V1-V3 or V3-V4 *16S* rRNA gene diversity profiling assays (Fig. 3b). Multivariable analysis via non-metric MDS (nMDS) showed groupings of samples based on the method of DNA isolation used, regardless of the *16S* rRNA gene diversity profiling assay used (Fig. 4a, b). Plotting vectors of the bacterial genera revealed alignment of samples with nMDS distribution with *Borrelia* and *Coxiella* (Fig. 4a, b). Focusing on the bacterial genus and class level of the microbiome, ANOSIM showed a significant difference in the bacterial community composition between those recovered using Method 1 compared to Method 2 (Fig. 4). Using a histogram of permutations, the observed R was outside the permutation distribution of the test statistics R under the null hypothesis from OTUs to class, and from OTU to order for both V1-V3 and V3-V4 *16S* rRNA gene diversity profiling assays, respectively (Fig. 4c, d). There were no significant differences between the individual ticks’ microbiomes at any taxonomical level, with the observed R close to the permutated distribution (Fig. 4c, d). Tukey’s multiple comparisons tests found significant levels of bias between Method 1 and Method 2 at all taxonomic levels, with the exception of phyla (Fig. 3a). Although not significant (Tukey’s multiple comparisons tests, *P* > 0.05), Method 1 found ~0.3–4.8% more reads representing *Borrelia* spp. at both V1-V3 and V3-V4 *16S* rRNA hypervariable regions than Method 2.

**Table 1:** Bacterial diversity measures for the Goanna tick *Bothriocroton undatum*

	S	N	H′	1-λ’
V1-V3 16S rRNA gene				
Method 1	57	12.16	0.1303	0.03108
Method 2	79	16.94	0.122	0.02785
Method 1 + 2	126	27.14	0.133	0.02947
V3-V4 16S rRNA gene				
Method 1	82	17.59	0.133	0.03071
Method 2	87	18.67	0.1479	0.03601
Method 1 + 2	124	26.71	0.148	0.03337

*Abbreviations*: *S* total number of species (OTUs), *N* species richness (Margalef), *H′*, Shannon index with logs to the base e, *1-λ’* Simpson index

### Method 1 with V1-V3 *16S* rRNA gene diversity profiling assay demonstrates capacity for *Borrelia* detection and identification

**Fig. 5:**
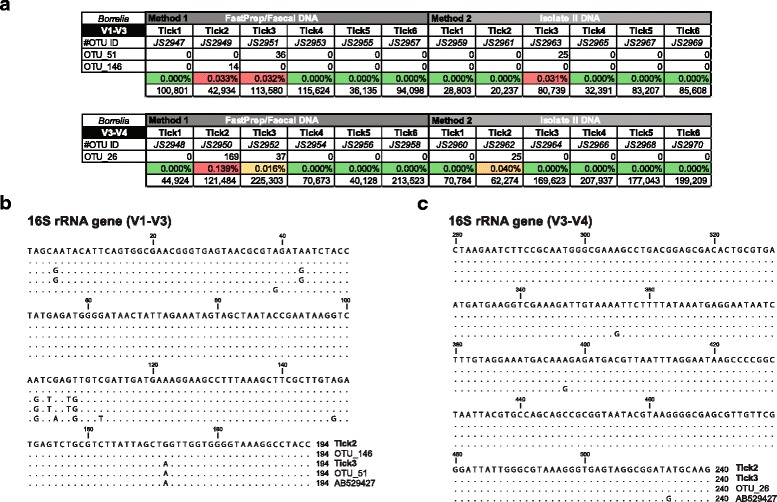
Molecular identification of reptile-associated *Borrelia* species in the Goanna tick (*Bothriocroton undatum*) from Sydney, Australia. **a** Summary table showing the V1-V3 and V3-V4 *16S* rRNA gene diversity profiling assay reads for *Borrelia* OTUs within each tick sample (Tick 1–6) recovered using either Method 1 or Method 2. The table includes the total number of high quality reads, the proportion of *Borrelia* reads, as well as the library identifier (JS2948-JS2970). **b**, **c** Multiple sequence alignment of the overlapping *Borrelia* OTU region of the V1-V3 (**b**) and V3-V4 (**c**) *16S* rRNA gene sequence, with *Borrelia* sp. *16S* rRNA gene sequence amplified from Tick2 and Tick3. Identical residues with the top reference are indicated by dots. Both *Borrelia* spp. Tick2 and Tick3 *16S* rRNA gene sequences are indistinguishable within the V3-V4 *16S* rRNA hypervariable region

The V1-V3 and V3-V4 *16S* rRNA gene diversity profiling assays revealed two *Borrelia* OTUs (V1-V3: OTU_51 and OTU_146), and one *Borrelia* OTU (V3-V4: OTU_26), respectively (Fig. 5a). Using Method 1, two ticks (33%, 2/6, Tick2 and Tick3) were positive for *Borrelia*, with the V3-V4 *16S* rRNA gene diversity profiling assay recovering identical OTU_26 (240-nt) in both ticks. The V1-V3 *16S* rRNA gene diversity profiling assay was able to detect OTUs in both Tick2 and Tick3 (V1-V3: OTU_51 and OTU_146, pairwise 96.7%, 8/240 identity). Method 2 only recovered *Borrelia* in Tick3 (V1-V3: OTU_51), matching the OTU using DNA extracted with Method 1. Using Method 2, Tick2 did not have any detectable *Borrelia* sequences, although it did have the lowest sequence depth of the examined ticks (20,237 sequence raw reads, Fig. 5a).

### Detection of two new reptile-associated *Borrelia* spp. in *Bothriocroton undatum* from Australia

Two tick samples (33%, 2/6, Tick2 and Tick3) analysed with the *16S* rRNA gene diversity profiling assays yielded ~1250-nt *Borrelia 16S* rRNA gene specific amplification products using DNA obtained with both Method 1 and Method 2. DNA sequencing revealed an unambiguous *16S* rRNA gene (1167-nt) sequence that was identical between amplicons from Method 1 and Method 2. The two *16S* rRNA gene sequences (Tick2 and Tick3) were 98.5% (17/1167) identical to each other. When comparing data from the V1-V3 *16S* rRNA gene diversity profiling assay and the conventional PCR, *16S* rRNA gene sequences obtained from Tick2 and Tick3 demonstrated 100% identity with OTU_146 (194/194) and OTU_51 (194/194), respectively (Fig. 5b). OTU_26 from the V3-V4 *16S* rRNA gene diversity profiling assay was 100% identical (240/240) with both Tick2 and Tick3 sequences obtained through conventional PCR (Fig. 5c).

The nested 23S rRNA gene PCR performed on Tick1–6 revealed the presence of *Borrelia* spirochetes in Tick2 (from DNA isolated using both Method 1 and Method 2, 222-nt product), while the remaining five ticks were PCR negative.

We subsequently inquired into the presence of *Borrelia* spp. in the remaining *B. undatum* ticks (Tick7–25). DNA was isolated using Method1 and subjected to the ~1250-nt *Borrelia 16S* rRNA gene specific conventional nested-PCR assay. A single DNA from Tick14 resulted in positive PCR amplification and the *16S* rRNA gene sequence was 100% identical to *Borrelia* sp. Tick3 (1167/1167) *16S* rRNA gene sequence.

In summary, three tick samples (12%, 3/25, Tick2, Tick3 and Tick14) yielded two distinct ~1250-nt *Borrelia* spp. *16S* rRNA gene specific amplification products. We tentatively named these two *Borrelia* sequences *Borrelia* sp. Tick2 and *Borrelia* sp. Tick3/Tick14.

**Fig. 6:**
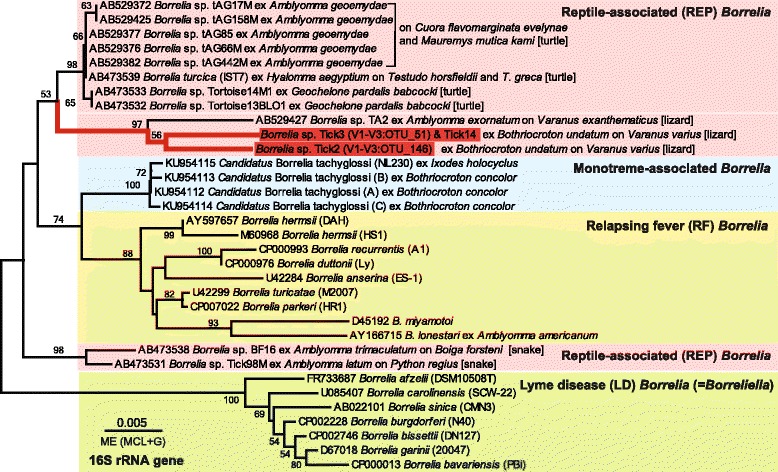
The evolutionary history of the reptile associated *Borrelia 16S* rRNA gene sequences. The tree was inferred using the Minimum Evolution (ME) method, with evolutionary distances computed using the Tajima-Nei method (TN), and the rate variation among sites modelled with a gamma distribution (+G, shape parameter = 0.28) in MEGA7. The percentage of replicate trees in which the associated taxa clustered together in the bootstrap test (500 replicates) are shown next to the branches. The tree is drawn to scale, with branch lengths in the same units as those of the evolutionary distances used to infer the phylogenetic tree. The analysis involved 33 nucleotide sequences. All positions containing gaps and missing data were eliminated. There were a total of 1075 positions in the final dataset. Accession numbers and species name and strain are depicted on the right. Colour coded major clades within *Borrelia* species include the monophyletic Lyme disease *Borrelia* (=*Borreliella*), the monophyletic Relapsing fever *Borrelia*, the monotreme-associated *Borrelia*, and the polyphyletic reptile-associated *Borrelia,* including turtle, lizard and snake groups

Multiple sequence alignment and phylogenetic analysis of *16S* rRNA gene sequences from representative *Borrelia* spp. with Tick2 and Tick3/Tick14 sequences revealed 98.5% (18/1167) and 98.2% (21/1167) identity with *Borrelia* sp. TA2 (AB529427) within a monophyletic clade (Fig. 6). The phylogenetic analysis clearly (> 95% bootstrap support) resolved monophyly of Lyme disease *Borrelia*, Relapsing fever *Borrelia* spp. and the monotreme-associated “*Candidatus* B. tachyglossi” (Fig. 6). The recognised sequences and *B. turcica* within the reptile-associated *Borrelia* species form three paraphyletic clades according to their tick host: snake, turtle and lizard (Fig. 6). The *Borrelia* spp. Tick2 and Tick3/Tick14, with *Borrelia* sp. TA2 (AB529427), form the lizard clade of the reptile-associated *Borrelia* species.

### Detection of *Rickettsia* cf. *tamurae* in *Bothriocroton undatum* from Australia

**Fig. 7:**
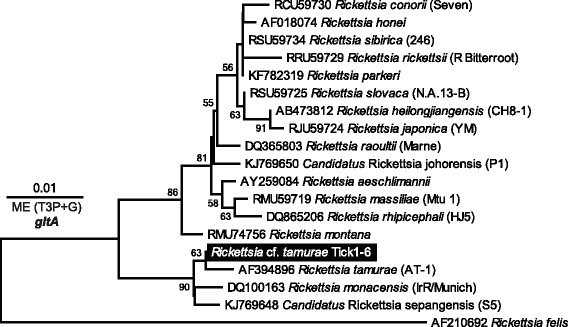
Phylogenetic tree of *Rickettsia* cf. *tamurae* in the Goanna tick (*Bothriocroton undatum*) from Sydney, Australia. The tree based on *gltA* gene sequences was inferred using the Minimum Evolution (ME) method with Tamura 3-parameter (T3P) distanced with gamma distribution (+G, shape parameter = 0.31) and calculated in MEGA7. Bootstrap support test (500 replicates) percentages are shown next to the branches. The tree is drawn to scale, with branch lengths in the same units as those of the evolutionary distances used to infer the phylogenetic tree. There were a total of 606 positions in the final dataset

All tick samples (6/6, Tick1–6) yielded ~650-nt *Rickettsia gltA* specific amplification products. DNA sequencing revealed unambiguous *gltA* sequences (606 nt), which were identical across all six ticks (Tick1–6). Multiple sequence alignment and phylogenetic analysis of *gltA* sequences from representative *Rickettsia* spp. revealed a high identity (99.7%, 2/606) to *Rickettsia tamurae* type strain AT-1 (AF394896). High identity (> 99%) at the *gltA* gene is indicative of species identity, therefore we tentatively consider our recovered species as *Rickettsia* cf. *tamurae* (Fig. 7). Negative controls remained negative throughout the diagnostic assay.

### Absence of *Coxiella burnetii* in *Bothriocroton undatum* from Australia

The *Coxiella burnetii* species specific PCR assay was negative for the six ticks examined (Tick1–6). The positive control for *C. burnetii* yielded a positive amplicon of expected 294 nt size. Negative controls remained negative throughout the diagnostic assay.

## Discussion

The study developed a methodology that resulted in the largest coverage of the tick microbiome, maximising the capacity to detect multiple *Borrelia* spp. within *B. undatum*, an endemic Australian tick species. The direct comparison of two DNA extraction protocols, each subjected to two *16S* rRNA gene diversity profiling assays, demonstrated bias associated with routine DNA extraction methods. The approach has enabled us to objectively propose an optimised workflow for the detection of *Borrelia* and *Rickettsia* species in ticks.

Analysis of the *16S* rRNA gene can be used to identify bacterial communities [39, 40]. The *16S* rRNA gene contains nine hypervariable regions: V1-V9 [39, 41]. Each region, however, shows a different capacity to define bacterial diversity, therefore no single region is able to differentiate all microbiota within the community of a tick [39, 42]. Our results are in line with these findings, as the V1-V3 *16S* rRNA gene diversity profiling assay identified a larger number of families, while the V3-V4 *16S* rRNA gene diversity profiling assay identified a larger number of genera. Diversity analyses showed no significant differences in the level of bacterial diversity between V1-V3 and V3-V4 *16S* rRNA hypervariable regions, or Method 1 and Method 2, despite an increasing trend in the diversity of V3-V4 *16S* rRNA hypervariable region and Method 2. Traditionally, the V3-V4 *16S* rRNA hypervariable region is preferred because diversity estimates for bacterial communities are often the highest [18]. Nevertheless, sequencing errors potentially result in an overestimation of the microbiome, increased diversity and bacterial taxa identification [18, 43]. With a single region being unable to identify all microbiota in a community, a wider span of the microbiome is achieved when multiple *16S* rRNA hypervariable regions are combined [18, 39, 44].

The design of the current study included obtaining near identical subsamples of each tick, providing us with the ability to demonstrate the effect of DNA isolation method as a significant source of variability. Previous research suggests that the type of sample collected, and DNA extraction protocol implemented during microbiome studies, can introduce variability and bias on the resulting microbiota [18, 45]. It is imperative to use a standardised DNA extraction approach to demonstrate microbiome associations with biotic factors. In fact, infections of *B. burgdorferi* in *Ixodes* spp. ticks were found to be significantly higher when sourced from woodlands as opposed to pastures, demonstrating the geographical effect on the presence of pathogenic bacteria in ticks [46]. Furthermore, the location and sex of *Ixodes* spp. has been shown to impact on the final microbiota identified, providing additional strength to our approach [25].

We demonstrated the presence of bacterial detection bias between two different approaches to DNA extraction (Method 1 and Method 2). These results conflict with those of Cruaud et al. [47] who argued that different DNA extraction methods lead to similar estimation of microbial diversity. Similarly, Rubin et al. [22] suggested that different DNA extraction protocols will only influence the ability, or lack thereof, of the microbiota to be sequenced, rather than impacting on the community recovered. Our results are in agreement with Vishnivetskaya et al. [45] who demonstrated that different DNA extraction kits can impact on the final interpretation of the microbiome. Evaluation of bias should be a prerequisite for any microbiome study because of the above noted controversies, which show the strength of the effect of DNA isolation approach on the subsequent microbiome sequenced.

For over 25 years there has been an ongoing debate on the presence of Lyme disease agents [*Borrelia burgdorferi* (*s.l.*)] in Australia, fuelled by findings of *Borrelia* spp. of unknown pathogenicity within Australian ticks [8–10, 15, 16, 48]. The current study demonstrates two more distinct *Borrelia* species of unknown pathogenic potential, found in 12% (3/25) of tick samples and tentatively named *Borrelia* sp. Tick2 and *Borrelia* sp. Tick3/Tick14. Recently, an Australian endemic “*Candidatus* B. tachyglossi” was identified in a closely related tick species, *B. concolor* [9]. Australian ticks are not free from *Borrelia* spp., yet the impacts of their presence on human and animal health remains unknown. Adopting a validated approach that has demonstrated the capacity to detect and differentiate *Borrelia* species should be applied across Australian ticks. Our results allow us to conclude that Method 1 combined with the V1-V3 *16S* rRNA hypervariable region results in the greatest capacity for *Borrelia* spp. detection.

*Borrelia* sp. Tick2 and *Borrelia* sp. Tick3/Tick14 both had a 98.2% similarity with *Borrelia* sp. TA2, a species previously identified within *Amblyomma geomydae* in Japan [49]. Takano et al. [49] found *Borrelia* sp. TA2 to be closely related to *B. turcica*, a reptile-associated (REP) *Borrelia* species. Because *Borrelia* sp. TA2, *Borrelia* sp. Tick2 and *Borrelia* sp. Tick3/Tick14 are monophyletic and were all sourced from hosts of the reptile genus *Varanus*, we believe that we have found two more REP *Borrelia* species that contribute to the ‘lizard’ clade.

In comparison to *Borrelia* spp., there is strong evidence for the presence of potentially pathogenic *Rickettsia* spp. within Australian ticks [4–7, 13]. Our study is in agreement with previous research, as our samples contained a high proportion (98–99%) of *Rickettsia* cf. *tamurae* (12/12, 100%). *Rickettsia* cf. *tamurae* is a member of the Spotted Fever Group Rickettsia, previously of unknown pathogenicity in humans [50]. However, in 2011 Imaoka et al. [51] described one of the first cases of human rickettsiosis in Japan caused by *R. tamurae* strain AT-1. In 2015, Kho et al. [52] further identified *R. tamurae* within *Amblyomma* ticks sourced from wild *Python molurus* in Malaysia. The current study has now identified *R. tamurae* within *B. undatum*, an endemic species of Australian tick. However, as our samples were only 99.7% similar to *R. tamurae* strain AT-1, is it possible that we have identified a new *R. tamurae* strain. Furthermore, when combined with previous research, our findings suggest the potential of this pathogenic bacteria to be distributed worldwide [49, 51, 52].

## Conclusions

The aim of the current study was to assess which combination of DNA extraction method with *16S* rRNA gene diversity profiling assay would give the greatest bacterial diversity, least amount of bias and greatest capacity for identification of *Borrelia* spp. and *Rickettsia* spp. We were able to establish an approach that satisfies all three aims, as well as demonstrate the presence of *Borrelia* spp. DNA in tick extracts. We have developed a molecular workflow capable of targeting *Borrelia* and *Rickettsia* spp., although we have limited insight on the origin of the bacteria identified and their pathogenic potential for human or animals.

## Additional files

Additional file 1: Table S1. Summary of tick samples used. **Table S2.** Summary of Qubit DNA concentrations and qPCR bacterial numbers estimates. **Table S3.** Summary of DNA sequencing of bacterial *16S* rRNA gene. (XLSX 18 kb)
